# Targeting zinc homeostasis to combat *Aspergillus fumigatus* infections

**DOI:** 10.3389/fmicb.2015.00160

**Published:** 2015-02-27

**Authors:** Rocío Vicentefranqueira, Jorge Amich, Paris Laskaris, Oumaima Ibrahim-Granet, Jean P. Latgé, Héctor Toledo, Fernando Leal, José A. Calera

**Affiliations:** ^1^Instituto de Biología Funcional y Genómica, Centro Mixto del Consejo Superior de Investigaciones Científicas y Universidad de Salamanca, Salamanca, Spain; ^2^Departamento de Microbiología y Genética, Universidad de Salamanca, Salamanca, Spain; ^3^Unité de Recherche Cytokines and Inflammation, Institut Pasteur, Paris, France; ^4^Unité des Aspergillus, Institut Pasteur, Paris, France

**Keywords:** *Aspergillus fumigatus*, zinc homeostasis, fungal pathogenesis, zinc transporters, transcription factors, drug discovery

## Abstract

*Aspergillus fumigatus* is able to invade and grow in the lungs of immunosuppressed individuals and causes invasive pulmonary aspergillosis. The concentration of free zinc in living tissues is much lower than that required for optimal fungal growth *in vitro* because most of it is tightly bound to proteins. To obtain efficiently zinc from a living host *A. fumigatus* uses the zinc transporters ZrfA, ZrfB, and ZrfC. The ZafA transcriptional regulator induces the expression of all these transporters and is essential for virulence. Thus, ZafA could be targeted therapeutically to inhibit fungal growth. The ZrfC transporter plays the major role in zinc acquisition from the host whereas ZrfA and ZrfB rather have a supplementary role to that of ZrfC. In addition, only ZrfC enables *A. fumigatus* to overcome the inhibitory effect of calprotectin, which is an antimicrobial Zn/Mn-chelating protein synthesized and released by neutrophils within the fungal abscesses of immunosuppressed non-leucopenic animals. Hence, fungal survival in these animals would be undermined upon blocking therapeutically the function of ZrfC. Therefore, both ZafA and ZrfC have emerged as promising targets for the discovery of new antifungals to treat *Aspergillus* infections.

## INTRODUCTION

Zinc is, after magnesium, the second most widespread metal present in enzymes belonging to all six major functional classes (Andreini et al., 2008). In addition, zinc ions are structural components of DNA binding domains of many transcription regulators and are required for their proper folding and binding to DNA. Consequently, zinc is essential for a wide variety of biochemical processes, for the adequate regulation of gene expression, and for cellular growth and development. Thus, when the cellular zinc content is lower than the “zinc quota,” i.e., the total amount of zinc required for a cell to grow optimally (Outten and O’Halloran, 2001), cell growth stops. In contrast, cells become intoxicated when the cellular zinc content exceeds an upper threshold. Like all organisms, the filamentous fungus *Aspergillus fumigatus* regulates tightly zinc homeostasis to maintain its own zinc quota. However, unlike most saprophytic fungi, *A. fumigatus* has different biological traits that turn it into an opportunistic pathogen (Tekaia and Latge, 2005), including its capacity to uptake zinc from host tissues (Amich et al., 2014). Thus, it is able to invade the lungs of susceptible individuals and causes invasive pulmonary aspergillosis (IPA), whose mortality rate may reach up to 90% depending on the host’s immune status (Kousha et al., 2011). One of the reasons for this high mortality rate is the low efficiency of the antifungal drugs currently in use to stop rapidly fungal growth. In this regard, we propose both regulation of zinc homeostasis and zinc acquisition as ideal therapeutic targets for the development of a next generation of antifungals, as an alternative to the classical antifungals that target either cell wall or ergosterol biosynthesis.

## ZINC AVAILABILITY AND MYCELIAL GROWTH OF *Aspergillus fumigatus*

The primary ecological niche of *A. fumigatus* is soil, where it grows as a saprophyte on organic decaying matter. In soils zinc is found in solution as free ions (Zn^2+^ and ZnOH^+^) and/or forming organic zinc complexes, as exchangeable zinc adsorbed to solid surfaces and forming insoluble complexes with other minerals (Alloway, 2008). The concentration of zinc in the soil liquid phase depends upon several factors but the ones that most strongly influence zinc solubility in soils are pH and moisture. Thus, although the concentration of soluble zinc in most soils ranges from 0.06 to 4.2 μM, in very acid soils it may exceed 110 μM (Alloway, 2008). Only the exchangeable zinc pool (i.e., the soluble zinc pool and the one that can be easily desorbed or released from soil particles) is available for plants and microorganisms. In addition, soils are dynamic environments that undergo wide fluctuations in many of their chemical and physical parameters that influence zinc availability. This explains why saprophytic soil inhabitants, particularly those adapted to grow in a wide range of pH values (e.g., *A. fumigatus*), are well equipped to face variations in soil zinc availability.

*Aspergillus fumigatus* has many genes encoding both zinc importers and exporters. The predicted role in zinc homeostasis of most of these transporters is based on what is known about similar proteins in other fungi, plants, and bacteria (Amich and Calera, 2014). The regulation of zinc homeostasis in *A. fumigatus* is mediated by the master zinc-responsive transcription factor ZafA (Moreno et al., 2007). In addition, the ZafA activity is further modulated in a pH-dependent manner likely influenced by the PacC transcriptional regulator (Amich et al., 2009, 2010). Thus, ZafA induces the transcription of *zrfA* and *zrfB* more strongly in acidic than in alkaline zinc-limiting media. In contrast, ZafA induces much more strongly the transcription of *zrfC* in alkaline than in acidic zinc-limiting media (Vicentefranqueira et al., 2005; Amich et al., 2010).

Although *A. fumigatus* is primarily a saprophytic fungus, it can grow also as a parasite within a susceptible, immunosuppressed animal host. Unlike soils, where zinc availability may change dramatically as a result of weather, leaching, animal activities, and/or human interventions, living environments provide homeostatic conditions in which zinc is tightly bound to zinc-binding proteins, such that the concentration of labile zinc in host tissues (i.e., the pool of free Zn^2+^ ions in both soluble and exchangeable form) is in the nanomolar range (Iyengar and Woittiez, 1988). Thus, zinc availability in living tissues is maintained constantly too low to support a sustained growth of most microorganisms, unless they have evolved mechanisms to overcome zinc starvation during their saprophytic growth that could be adapted for parasitic growth. In this regard, *A. fumigatus* takes advantage of the zinc transporters ZrfA, ZrfB, and ZrfC to grow in the slightly alkaline and extreme zinc-limiting environment provided by the host.

## ZINC ACQUISITION AND VIRULENCE IN *A. fumigatus* AND OTHER FUNGAL PATHOGENS

**FIGURE 1:**
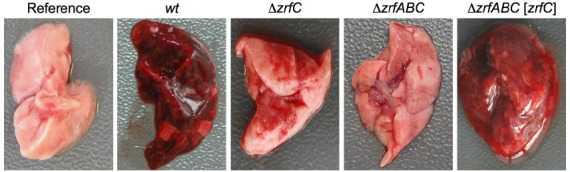
**Lungs from immunosuppressed mice infected with different *A. fumigatus* strains.** The mice were immunosuppressed using a leucopenic regime and inoculated intranasally with 10^5^ conidia of the AF14 (wild-type), AF54 (Δ*zrfC*), AF721 (Δ*zrfA*Δ*zrfB*Δ*zrfC*), and AF731 (Δ*zrfA*Δ*zrfB*Δ*zrfC[zrfC]*) strains, as described in Amich et al. (2014). Mice were sacrificed after 4 days post-inoculation and the left lung was excised and photographed. The lungs from mice infected with the wild-type and Δ*zrfABC[zrfC]* strains showed the greatest signs of pulmonary infarction. In contrast, the lungs from mice inoculated with the Δ*zrfC* strain showed a very low degree of infarction. The lungs from mice inoculated with the Δ*zrfABC* strain appeared healthy similar to that from a non-inoculated mouse used as reference.

The relationship between zinc homeostasis and virulence of human fungal pathogens was shown for the first time in *A. fumigatus*. The ZafA-mediated regulation of zinc homeostasis is essential for *A. fumigatus* growth within a host but dispensable for it to grow as a saprophyte in zinc-replete media (Moreno et al., 2007). The avirulence of a Δ*zafA* mutant of *A. fumigatus* primarily resides in its inability to obtain zinc from the host, which results in a zinc shortage that stops germ tube elongation. This is consistent with the avirulence of a Δ*zrfA*Δ*zrfB*Δ*zrfC* strain (Amich et al., 2014), which lacks the three major downstream targets of ZafA. However, the ZrfA and ZrfB acidic zinc transporters and the ZrfC alkaline zinc transporter contribute differentially to fungal virulence as shown by the appearance of lungs excised from mice infected with the Δ*zrfA*Δ*zrfB* and Δ*zrfC* mutants (Figure 1). The ZrfC protein plays a major role in obtaining zinc from living tissues and sustaining fungal growth within them, whereas the acidic transporters ZrfA/B have a supplementary function in zinc acquisition from the host. The importance of ZrfC for fungal virulence relies on its long extracellular N-terminus, which has four putative zinc-binding motifs and that is absent in the acidic transporters (Amich et al., 2010, 2014). Indeed, in terms of fungal virulence the zinc uptake activity of the ZrfC protein without its N-terminus equals to that of ZrfA and ZrfB together (Amich et al., 2014). ZafA-like regulatory systems have also been described in and related to the virulence of the pathogenic fungi *Cryptococcus gattii*, *Candida albicans*, and *Paracoccidioides brasiliensis*, although in these latter two microorganisms this has not yet been experimentally confirmed (Nobile et al., 2009; Schneider et al., 2012; Parente et al., 2013). These fungal pathogens have genes encoding proteins similar to ZrfA/B and ZrfC, although the N-terminus of the *C. gatti* ZrfC-like protein (XP_003195293) is very different from that of ZrfC and the *C. albicans* (XP_715421) and *P. brasiliensis* (XP_010761875) ZrfC-like proteins. In either case, the roles of these transporters for virulence have not been investigated.

## ZINC-BASED STRATEGIES DEPLOYED BY PHAGOCYTES TO INHIBIT MICROBIAL GROWTH

**FIGURE 2:**
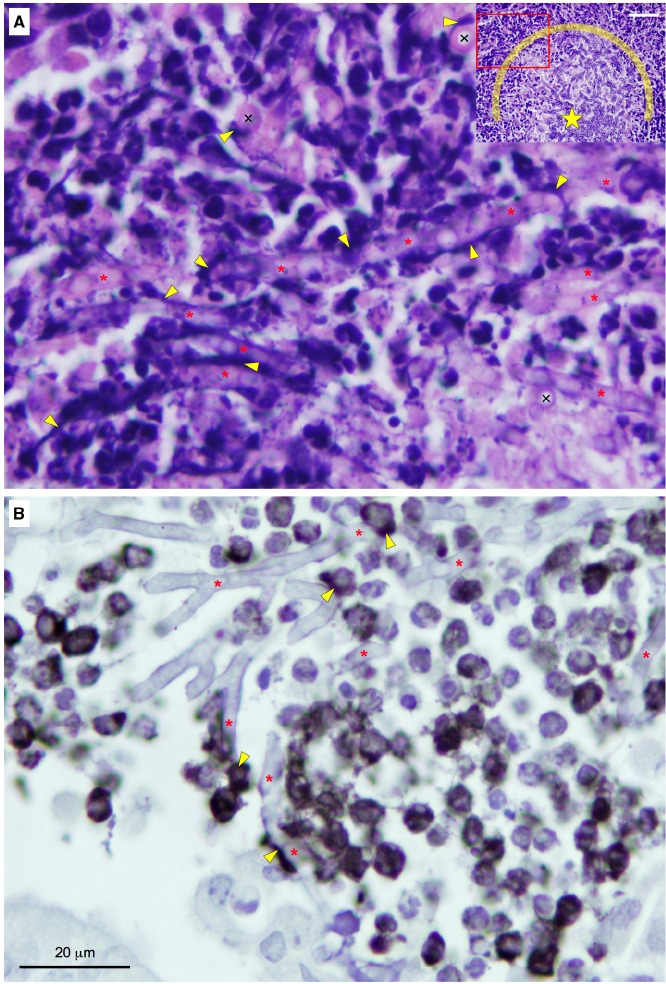
**Detailed observation of the edge of *A. fumigatus* abscesses heavily infiltrated with immune cells in lung sections from non-leucopenic mice infected with a wild-type strain.** The mice were euthanized after 4 days post-inoculation. The lungs were fixed and processed for histology and immunocytochemistry, as described in Amich et al. (2014). The slides were stained with H&E **(A)** or immunostained using an antibody against the S100A8 component (calgranulin A) of calprotectin **(B)** and observed at the highest magnification using with a 100× objective. In H&E-stained slides of lungs infected with filamentous fungi is usually hard to distinguish between scattered hyphae and the surrounding lung tissue. However, in abscesses with a high fungal load, hyphae are easily recognized from the surrounding lung tissue in H&E-stained slides. In panel **(A)** is shown in great detail the edge of an abscess with a heavy fungal load. Lengthwise hyphae are indicated with red asterisks and cross-sectioned hyphae are indicated with the “×” symbol. Yellow arrowheads indicate haematoxylin-positive thread-like structures and patches. The hyphae more heavily covered with haematoxylin-positive material were typically located at the outlying region of the infectious foci (see the inset image as a reference) and showed both a degree of vacuolization higher and a diameter wider than those of hyphae located toward the middle of the infectious foci (indicated with a star in the inset), which probably anticipates their lysis. In the inset is shown about half of the infectious focus chosen to take the picture (inset bar = 50 μm). The red rectangle in the inset delimits the enlarged image showed in panel **(A)**. The area enclosed by the semicircle around the star delimits the part of the infectious focus with the heaviest fungal load. In panel **(B)** calprotectin was immunodetected either associated to immune cells (most likely pDCs and/or neutrophils) or laid out over hyphae.

The regulation of zinc homeostasis is especially relevant for pathogens because the amount of labile zinc in host tissues is very low. Accordingly, mammals have evolved the capacity to inhibit microbial growth in their tissues by zinc starvation, as part of a broader defense mechanism termed “nutritional immunity” (Hood and Skaar, 2012). Two different strategies to sequester labile zinc have been described in mammals thus far. One of them is directed to inhibit the growth of extracellular pathogens and relies upon releasing of calprotectin (CP) by neutrophils in abscesses (Corbin et al., 2008). The other one operates in activated macrophages infected with intracellular pathogens and involves the binding of zinc to metallothioneins (MTs; Subramanian Vignesh et al., 2013). However, an excess of zinc is also noxious for pathogens, probably because it reacts with the –SH groups of proteins. Thus, it is not surprising that mammals had evolved also an antimicrobial strategy based upon zinc poisoning of microbes enclosed in macrophage endosomes (Botella et al., 2011). These defense strategies against pathogens deployed by mammals are consistent with the notion that zinc acquisition and zinc detoxification are virulence attributes of bacterial and fungal pathogens. It might be possible that both MTs and zinc poisoning play a role in macrophages against *A. fumigatus*, as they do respectively against *Histoplasma capsulatum* (Subramanian Vignesh et al., 2013) and *Mycobacterium tuberculosis* (Botella et al., 2011). However, the effects of MTs and zinc poisoning on conidia germination inside the macrophages have not been investigated. In contrast, it has been reported that CP is the major component of the extracellular traps released by neutrophils (NETs) as a defense against *C. albicans* (Urban et al., 2009). NETs are also produced against *A. fumigatus* both *in vitro* and *in vivo* (Bruns et al., 2010; McCormick et al., 2010). More recently it has been shown that CP can reduce the growth capacity *in vitro* of *A. fumigatus* and that the ability to grow of this fungus in the presence of CP under alkaline Zn/Mn-limiting conditions relies on the function of ZrfC (Amich et al., 2014). The only leukocytes infiltrated into *A. fumigatus* abscesses in immunosuppressed non-leucopenic mice that are able to produce CP are the polymorphonuclear neutrophils (PMNs) and plasmacytoid dendritic cells (pDCs; a subtype of DCs). PMNs produce huge amounts of CP that is released in NETs following their lysis (Urban et al., 2009) whereas pDCs synthesize and transport CP to their surface upon activation (Ramirez-Ortiz et al., 2011). In fungal abscesses heavily infiltrated with leukocytes we have observed patches and thread-like structures stained intensely with hematoxylin (Figure 2A). These structures, which might correspond to chromatin present in DCs, monocytes, PMNs, and/or NETs, are either spread out on the surface of hyphae or enveloping them. Interestingly, the observation at high magnification of infiltrated abscesses immunostained for CP indicated that this was most frequently detected in leukocytes located in close proximity to hyphae or attached to them (Figure 2B). This suggests that CP may create a Zn/Mn-deprived microenvironment around fungal cells to restrict their growth at the time that *A. fumigatus* manages to overcome the inhibitory effect of CP through the action of the ZrfC. Thus, ZrfC has a dual role in fungal virulence: it is needed to mediate zinc uptake and to counteract the inhibitory effect of CP in abscesses.

## THE REGULATION OF ZINC HOMEOSTASIS AND ZINC ACQUISITION AS IDEAL TARGETS FOR ANTIFUNGAL DISCOVERY AND DEVELOPMENT

The fact that host mammals are able to inhibit microbial growth either by intoxicating pathogens with zinc or by limiting the access of pathogens to the host zinc pool have led us to presume that these strategies could be imitated to control therapeutically the growth of pathogens in a host.

The lack of knowledge about whether the host immune cells use zinc poisoning to prevent or combat *A. fumigatus* infections and, in this case, whether the fungus has countermeasures to protect itself from it, does not allow us to envision to date a specific therapeutic approach to mimic it. In contrast, what is known to date about zinc homeostasis is enough for us to propose that a therapeutic approach based on preventing the access of the fungus to host zinc would be deleterious for fungal cells because fungal growth depends on a constant intake of zinc and fungal zinc depletion increases the synthesis of harmful reactive oxygen species (reviewed in Eide, 2011). Thus, any compound that interfere intra or extracellularly with zinc homeostasis would predictably inhibit fast and efficiently the fungal growth within host tissues.

The major challenge to treat IPA by targeting zinc homeostasis relies on the identification of the targets and the discovery and development of specific antifungal compounds. Regarding the first point, we propose the ZrfC transporter and ZafA transcription factor as targets. Nutrient transporters are currently considered as new promising therapeutic targets (Slavic et al., 2011; Prati et al., 2014). ZrfC has a long N-terminus that has been predicted to be located toward the extracellular side of the plasma membrane and, hence, that would be readily accessible to specific inhibitors (Amich et al., 2010). In addition, it would be expected that any interference with the function of the N-terminus of ZrfC had the same effect as inactivation of the whole protein in terms of both fungal growth and virulence (Amich et al., 2014). However, the inactivation of ZrfC is partially compensated by an increase in the expression level of the acidic transporters (ZrfA and ZrfB; Amich et al., 2014), which is most likely mediated by ZafA. This indicates that to block efficiently fungal growth it would be required to inhibit either ZrfA/B or ZafA in addition to ZrfC. By taking advantage of the key role of ZafA in the homeostatic and adaptive responses to zinc deprivation, we consider that ZafA would be an alternative target to ZrfA/B. However, to inhibit or reduce noticeably the function of any transcription factor it would be required to disrupt specifically either a DNA–protein or a protein–protein interaction. To disrupt the interaction of ZafA with DNA we should target their C_2_H_2_ zinc fingers. However, given the highly conserved C_2_H_2_ structure of the zinc fingers in many transcription factors from fungi to humans have led us to think that is very unlikely to find a drug able to bind selectively to the zinc fingers of ZafA without interfering with human transcription factors. Thus, we propose to target the ZafA transactivation domains to disable the specific interaction of ZafA with the transcriptional machinery. For instance, this mechanism of action has been reported for triptolide, which is a natural compound that inhibits the transactivating function of the human HSF1 transcription factor (Westerheide et al., 2006).

Importantly, the ZafA- and ZrfC-like proteins are distributed exclusively among fungi and no orthologs have been found in mammals. In fact, the human MTF-1 protein (reviewed in Gunther et al., 2012), which might be considered functionally analogous to ZafA, is structurally unrelated to ZafA. Moreover, the hMTF-1 regulator functions in an opposite manner to that of ZafA. Thus, hMTF-1 induces gene expression under zinc-replete conditions whereas ZafA induces gene expression under zinc-limiting conditions. On the other hand, ZrfC is very distantly related to each human ZIP protein. Indeed, it is very difficult to infer a functional similarity of ZrfC with any of the 14 human ZIP proteins (reviewed in Cousins et al., 2006), which suggests that the mechanism of action of ZrfC might be disrupted without disturbing the action of any hZIP.

The chemical compounds able to disrupt zinc homeostasis in the fungus growing within the host could exert their effect by either chelating zinc or by inhibiting selectively the function of specific proteins that are essential to maintain zinc homeostasis. The use of chelating agents has been explored as an option for anticancer (Huesca et al., 2009; Lui et al., 2012; Zuo et al., 2012; Fatfat et al., 2014) and antimicrobial therapies (reviewed in Santos et al., 2012). Several studies have shown that the treatment with different zinc chelators kills different types of cancer cells, which suggest that zinc chelators may be theoretically useful for the treatment of different types of cancer (Torti et al., 1998; Hashemi et al., 2007; Huesca et al., 2009; Zuo et al., 2012). In contrast, most evidences about the reliability of using metal chelators for the treatment of *A. fumigatus* infections have arisen from the use of iron chelators either alone or combined with classical antifungal drugs (Zarember et al., 2009; Ibrahim et al., 2010; Leal et al., 2013). The only study to support the notion that zinc chelation might be useful to combat *A. fumigatus* arose from the finding that EDTA is an adjunct antifungal agent in a rodent model of IPA (Hachem et al., 2006). However, EDTA is a broad-spectrum chelator, so whether its antifungal effect is due to its capacity to chelate zinc and/or other metals is unknown. Nevertheless, the survival rate of mice with IPA increases significantly when they are treated with the zinc-specific chelator TPEN (unpublished data), which demonstrates that a compound able to interfere with zinc homeostasis can be useful as antifungal drug. However, available scientific evidences do not support claims that chelation therapies provide a safe treatment for either cancer or infectious diseases. In this regard, it has been reported that iron chelation has the potential risk of producing undesired side effects in human patients by altering the normal iron homeostasis (Kontoghiorghes et al., 2010). Likewise, the American Cancer Society has warned about the harmful side effects of chelation therapy. Moreover, loss of zinc can also lead to mutations in cells, which may actually increase the risk of cancer (Song et al., 2009). In summary, the reliability of chelation therapies remains too controversial as to be considered the best option (Nissen, 2013). Instead, the discovery of compounds that specifically block the function of fungal proteins required for counteracting host defense mechanisms and/or zinc acquisition (e.g., ZafA and ZrfC) appears more promising. For instance, it has been found recently an anti-mycobacterial compound that interferes with zinc homeostasis by inactivating the countermeasures used by *Mycobacterium* to protect itself of the zinc-mediated intoxication deployed by host phagocytes to kill it inside the phagosomes (Rybniker et al., 2014). We await that a screening to identify anti-*Aspergillus* compounds lead us to the discovery of new drugs to block the regulation of zinc homeostasis and zinc acquisition.

## CONCLUSIONS AND OUTLOOK

The regulation of zinc homeostasis by ZafA and zinc acquisition by ZrfC constitute highly promising therapeutic targets to combat *Aspergillus* infections and perhaps also other infections caused by fungal pathogens bearing similar targets. Furthermore, by targeting two different steps (regulation of zinc homeostasis and zinc acquisition) of the same biological process (zinc metabolism) with a specific combination of two drugs we could get two of the major achievements in the development of emerging antimicrobial therapies: (i) a synergistic interaction and, (ii) a significant reduction in the probability of evolving mutations that enhance the resistance of the fungus to any of these compounds (Cottarel and Wierzbowski, 2007). Finally, to prevent the unavoidable pleiotropic effect of chelators on host cells we propose that future efforts to develop inhibitors for homeostasis of specific metals in pathogens, including *A. fumigatus*, should be directed to the discovery of compounds that specifically block the function of microbial proteins required to regulate the homeostasis and/or acquisition of metals.

### Conflict of Interest Statement

The authors declare that the research was conducted in the absence of any commercial or financial relationships that could be construed as a potential conflict of interest.
